# Evaluation of Glucose-6-Phosphate Dehydrogenase stability in stored blood samples 

**DOI:** 10.17179/excli2015-604

**Published:** 2016-02-19

**Authors:** Norunaluwar Jalil, Raja Zahratul Azma, Emida Mohamed, Azlin Ithnin, Hafiza Alauddin, Siti Noor Baya, Ainoon Othman

**Affiliations:** 1 Department of Pathology, Universiti Kebangsaan Malaysia Medical Centre, Kuala Lumpur; 2 Department of Medical Laboratory Technology, Universiti Teknologi MARA, Puncak Alam, Selangor; 3 Department of Medical Sciences II, Faculty of Medicine, Universiti Sains Islam Malaysia, Kuala Lumpur

**Keywords:** G6PD stability, G6PD enzyme assay, OSMMR2000-D G6PD Assay Kit, EDTA, dried blood spot

## Abstract

Glucose-6-Phosphate Dehydrogenase (G6PD) deficiency is the commonest cause of neonatal jaundice in Malaysia. Recently, OSMMR2000-D G6PD Assay Kit has been introduced to quantitate the level of G6PD activity in newborns delivered in Universiti Kebangsaan Malaysia Medical Centre (UKMMC). As duration of sample storage prior to analysis is one of the matters of concern, this study was conducted to identify the stability of G6PD enzyme during storage. A total of 188 cord blood samples from normal term newborns delivered at UKMMC were selected for this study. The cord bloods samples were collected in ethylene-diamine-tetra-acetic acid (EDTA) tubes and refrigerated at 2-8 °C. In addition, 32 out of 188 cord blood samples were spotted on chromatography paper, air-dried and stored at room temperature. G6PD enzyme activities were measured daily for 7 days using the OSMMR2000-D G6PD Assay Kit on both the EDTA blood and dried blood samples. The mean value for G6PD activity was compared between days of analysis using Student Paired T-Test. In this study, 172 out of 188 cord blood samples showed normal enzyme levels while 16 had levels corresponding to severe enzyme deficiency. The daily mean G6PD activity for EDTA blood samples of newborns with normal G6PD activity showed a significant drop on the fourth day of storage (p < 0.005) while for samples with severely deficient G6PD activity, significant drop was seen on third day of storage (p = 0.002). Analysis of dried cord blood showed a significant reduction in enzyme activity as early as the second day of storage (p = 0.001). It was also noted that mean G6PD activity for spotted blood samples were lower compared to those in EDTA tubes for all days (p = 0.001). Thus, EDTA blood samples stored at 2-8 °C appeared to have better stability in terms of their G6PD enzyme level as compared to dried blood samples on filter paper, giving a storage time of up to 3 days.

## Introduction

Glucose-6-Phosphate Dehydrogenase (G6PD) is an enzyme in the hexose monophosphate oxidative pathway of the red blood cells. The G6PD enzyme maintains the production of the cofactor nicotinamide adenine dinucleotide phosphate-oxidase (NADPH). NADPH, on the other hand, is important for maintenance of reduced glutathione which involves in detoxifying toxic oxidative products in the body. Hence, G6PD deficiency results in a loss of this protective effect and leads to oxidative damage of the red cells. This occurs when the affected individuals are exposed to oxidative stress such as infections, certain drugs and foods. Most individuals with deficiency in G6PD enzyme are asymptomatic (Iranpour et al., 2003[9]) but may episodically suffer from acute haemolytic anemia. However, the most important complication of G6PD deficiency is severe neonatal jaundice which may lead to brain damage or even death.

G6PD deficiency is the commonest enzymopathy in human affecting approximately 400 million individuals worldwide and is an X-linked disorder. It has a wide geographical distribution and is highly prevalent in Africa, Mediterranean, Southeast Asia and Middle East (Ainoon et al., 2003[1]; Allahverdiyev et al., 2012[3]). In Malaysia, the overall incidence of G6PD deficiency among males is 3.1 %. It was reported to be more common among the Chinese and Malays as compared to Indians (Ainoon et al., 2003[2]). A national programme of mandatory screening for G6PD deficiency of all newborns has been instituted since early 1980s, employing the WHO recommended semiquantitative Fluorescent Spot Test (FST) because of the association with severe neonatal jaundice (Beutler and Mitchell, 1968[6]). However, the lack of sensitivity of the FST to detect individuals with moderate enzyme deficiency (activity > 20 % < 60 % of normal mean) (Reclos et al., 2000[12]; Minucci et al., 2008[10], Ainoon et al., 2003[1]) who are also at risk for severe haemolysis, has led us to introduce the quantitative G6PD enzyme assay method at our centre for routine screening of newborns (Ainoon et al., 2003[1]; Azma et al., 2010[4]). Being the sole hospital to offer G6PD assay as a service our center has become a referral laboratory and have been receiving a substantial number of samples from other hospitals including state hospitals. One of the problems faced by our clients is the effect of time and temperature on the enzyme activity of the samples during transportation (Castro et al., 2005[7]; Odoula et al., 2005[11]) which in turn may affect the accuracy and the consistency of the results from outside referred samples. Hence, this study was conducted to evaluate the stability of G6PD activity in-vitro and to identify the maximum storage time that a sample can be stored and to give a consistent reading on different methods of storage.

## Materials and Methods

One hundred eighty eight neonates (97 males; 91 females) born at the Universiti Kebangsaan Malaysia Medical Centre were selected within a period of 4 months study. The study was performed on EDTA cord blood samples received at the Haematology Laboratory, Department of Laboratory Diagnostics Service, UKMMC, for routine G6PD screening by FST. Informed consents were obtained from parents of newborns involved in the study. This study was approved by Institutional Human Research Committees of UKMMC and Universiti Teknologi MARA, Campus Puncak Alam. Red blood cell G6PD activity assays were performed on all the blood samples in the EDTA tubes. Out of the 188 cord blood samples, 32 cord blood samples spotted on chromatography paper and air-dried. The G6PD activity assay was done daily for seven consecutive days during which the EDTA cord blood, stored at 2-8 °C and the air-dried blood on chromatography paper, stored in a dark place at room temperature. G6PD assays were performed using OSMMR-D G6PD assay kit with haemoglobin normalization from R and D Diagnostics (Holargos, Greece) and enzyme activity measured on a spectrophotometer (Ultramicroplate Reader EL808, Bio Tek, Instruments) as previously described (Azma et al., 2010[4]). For the batch of control samples provided by the manufacrurer, a reference range of G6PD activity for the newborns was established as recommended (Azma et al., 2010[4]). The mean G6PD activity for the newborns with normal G6PD activity was 12.8 U/g Hb. The cut-off points for G6PD deficiency were established according to WHO classification and are as follows: 7.69 U/g Hb (60 % of normal mean), 2.56 U/g Hb for severe deficiency (20 % of normal mean activity) and 2.56 to 7.69 U/g Hb being the range for moderate deficiency (20 % - 60 % of normal mean activity) (Azma et al., 2010[4]).

### Statistical analysis

The statistical analysis was done using SPSS version 18.0 software. Daily mean of G6PD enzyme activity were analysed using Student Paired T-Test. P-value < 0.005 was considered statistically significant difference in mean of G6PD activity.

## Results

Of the 188 selected newborns, the ethnic distribution was Malays 72 % (n = 134), followed by Chinese 21 % (n = 40), Indians 3 % (n = 6) and others 4 % (n = 8). Others comprised of Iban, Kadazan, Kayan, Indonesia, Myanmar, Pakistan, Sudan and Thai origin. 

172/188 (91 %) samples were found to have normal G6PD activity and 16/188 (9 %) were severe deficient. Of the samples with normal G6PD activity, 124/172 (66 %) were Malays, 35/172 (19 %) Chinese and 6/172 (4 %) Indians. As for the samples with severely G6PD deficiency, 9 (5 %) were Malays, 5 (3 %) were Chinese and the remaining of 2 (1 %) were Kadazan and Myanmar.

**Figure 1:**
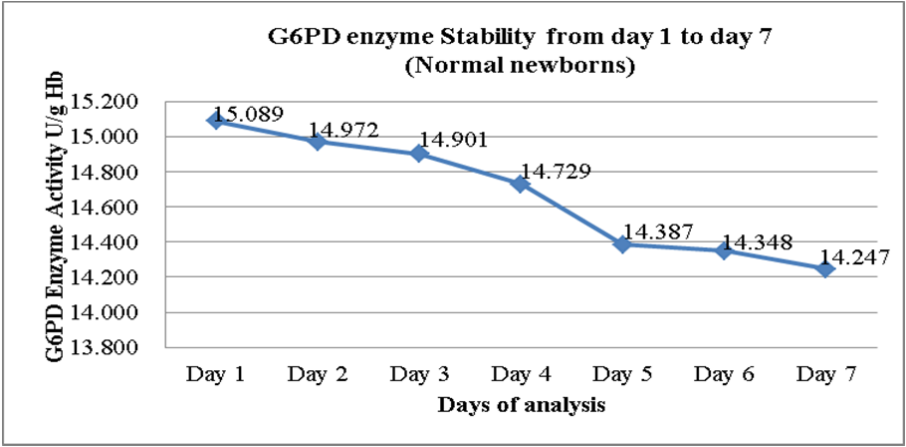
Mean G6PD activity in EDTA cord blood samples from neonates with normal G6PD Activity (n = 172)

The daily mean G6PD activity level for the 172 newborns with normal G6PD activity was tabulated in (Figure 1(Fig. 1)). There was slight drop in the enzyme activity after Day 1 and Day 2 but the reduction demonstrated were not statistically significant in the mean G6PD activity between Day 1 and Day 2, and also between Day 1 and Day 3 (p = 0.033 and 0.006 respectively) (Table 1(Tab. 1)). However, the mean G6PD activity showed statistically significant drop (p < 0.005) on the fourth day of storage and onwards. There was a statistically significant difference in the mean G6PD activity between Day 1 and Day 4, and the subsequent days (p < 0.005). The differences in the mean of G6PD activity between Day 1 and Day 7 were 0.842 U/g Hb (p < 0.005).

**Figure 2:**
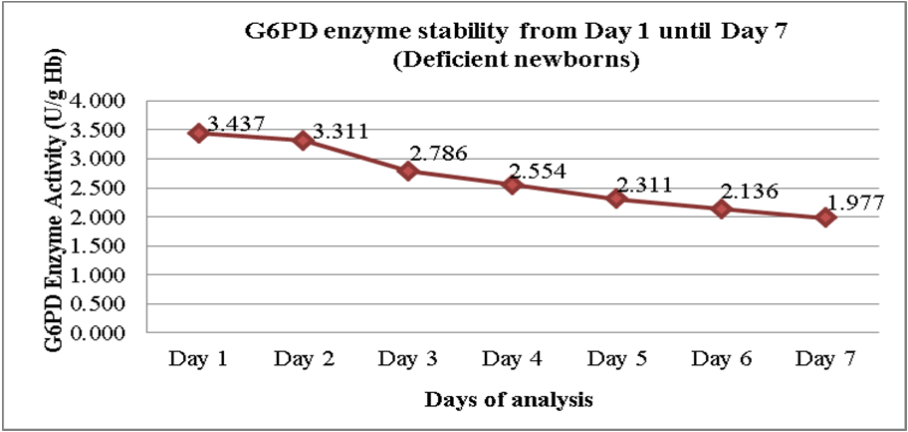
Mean G6PD activity in EDTA cord blood samples with deficient G6PD activity (n = 16)

Figure 2(Fig. 2) shows the daily mean G6PD activity in the cord blood samples of 16 newborns with G6PD deficiency. There was no statistically significant differences in the means G6PD activity between Day 1 and Day 2 (p = 0.327) (Table 2(Tab. 2)). However, there were statistically significant differences in the means G6PD activity of G6PD deficient newborns between Day 1 and Day 3, and the subsequent days (p < 0.005). The differences of the means G6PD activity between Day 1 and Day 7 was 1.459 U/g Hb (p < 0.005).

**Figure 3:**
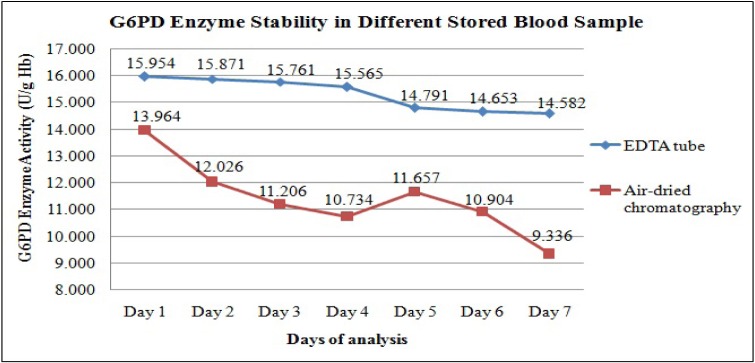
Comparison of daily mean G6PD activity between stored blood from EDTA tube and stored blood spots on chromatography paper (n = 32).

Figure 3(Fig. 3) shows the changes in the daily mean G6PD activities in the 32 cord blood spotted and air-dried on chromatography paper. The G6PD activity appeared to drop from Day 1 to Day 4 of analysis, but on Day 5, the activity was slightly increased before it dropped again on Day 6 and Day 7. The differences in the mean G6PD activity between Day 1 and Day 7 were 4.628 U/g Hb (p < 0.005). Statistically significant differences in G6PD activity were observed between Day 1 and Day 2, and onwards (p < 0.005) (Table 3(Tab. 3)).

## Discussion

Routine testing for G6PD deficiency by FST has been shown to miss substantial proportion G6PD deficient individuals who have moderate enzyme deficiency (Ainoon et al., 2003[1]). They were mostly female heterozygotes who were classified as partially deficient. However, they had been shown to be at risk to haemolyis and severe neonatal jaundice. The enzyme assay method for the quantitation of RBC G6PD activity was introduced in UKMMC as a service since 2010 to address this problem. Due to the scarcity of hospitals providing this service and the realization of the importance of G6PD activity measurement as part of strategy of prevention of severe neonatal jaundice associated with G6PD deficiency, UKMMC laboratory has offered this service to other hospitals. EDTA cord blood samples were currently transported from state hospitals to UKMMC. Most of the time it involves extended period of time, may exceed 24 hours due to long distance. Hence, inappropriate storage may affect the stability of G6PD enzyme and jeopardizing the accuracy of the measurement of the G6PD activity level. 

In the present study we found that G6PD activity measurement of cord blood samples in EDTA tube, when stored at 2-8 °C, began to show slight drop after Day 1. However, a significant drop was only seen on fourth day and the subsequent days for the samples from newborns with normal G6PD activity. There were statistically significant differences in the means of G6PD activity between Day 1 and Day 4 onwards p < 0.005 (Figure 1(Fig. 1) and Table 1(Tab. 1)). These findings seem to be consistent with the findings by Castro et al. (2005). In their works, they found that G6PD enzyme activity for blood samples in EDTA tube stored refrigerated at 2-8 °C gave consistent reading for G6PD activity up to 72 hours from time of collection. 

As for the severe G6PD deficiency group, the enzyme activity appeared to be reduced also seen after Day 1, but significant drop was detected much earlier as compared to the normal G6PD group. A significant drop was seen starting Day 3 onwards. It was shown that the means of G6PD activity on Day 1 was 3.42 U/g Hb where else its value on Day 3 was 2.78 U/g Hb (p = 0.002). It was also noted that within the storage period of seven days, the mean differences of G6PD activity between Day 1 and Day 7 was 1.459 U/g Hb. (p = 0.000). 

The established cut-off points for G6PD deficiency for this study was 7.69 U/g Hb (60 % of 12.8 U/g Hb; the normal mean activity) and 2.56 U/g Hb for severe deficiency (20 % of normal mean activity) while the range for moderate deficiency (20 % - 60 % of normal mean activity). The reference ranges were established earlier by Azma et al., 2014[5]. Thus, based on the current findings, if there was a reduction of mean G6PD activity by 1.459 U/g Hb after seven days of collection, partial deficient newborns might wrongly be diagnosed as severe G6PD deficiency. 

We have also explored the possibility of using air-dried cord blood samples spotted on the chromatography paper as a potential alternative method of collecting blood and transportation. The convenience of this method lies in the fact that blood samples could be stored at room temperature and transported in dry form. It will then provide a more practical way for transportation of cord blood samples from remote areas which may take days to reach UKMMC. However, the current study demonstrated that enzyme activity on dried blood samples showed a significant drop in G6PD activity as early as Day 2 of storage. There was statistically significant difference in the mean G6PD activity between Day 1 and Day 2 (13.9 U/g Hb vs 12.02 U/g Hb, respectively, p < 0.005). Similar findings were also described by Fujimoto et al. (2000[8]), suggesting reduced the G6PD enzyme stability in dried blood spot, probably due to the adversely affected by humidity and temperature.

A comparison on the G6PD enzyme stability between two different methods of collection and transportation was performed. We found that the mean G6PD activity of dried blood samples were lower than the mean G6PD activity for EDTA tube samples beginning even on Day 1 (13.96 U/g Hb vs 15.9 U/g Hb, p = 0.001) (Table 3(Tab. 3)). The G6PD activities for the dried blood samples were significantly lower than the activity for the EDTA tube samples on all the 7 days (Figure 3(Fig. 3)). Hence, our findings showed that the dried blood samples resulted in lower enzyme stability as compared to blood samples stored in EDTA tubes. In addition, the enzyme activity on air-dried blood spot chromatography paper fluctuated on the first five days of analysis, whiles that in EDTA tube consistently decreased over the time. The possibilities that could have contributed to these findings were due to pipetting technique. EDTA is the one of the recommended anticoagulants that can be used to preserve the G6PD enzyme in the erythrocytes. Previous study by Oduola et al. (2005[11]), showed that G6PD enzyme was stable for up 72 hours at 4 °C in EDTA tube, followed by ACD, CPD, sodium citrate and lithium heparin. 

## Conclusion

The OSMMR-D G6PD assay kit is rapid, easy to perform, less laborious and has good reproducibility. The maximum days of a cord blood sample that can be stored in EDTA tube and refrigerated at 2-8 °C to give a consistent reading for G6PD assay, was 3 days. Spotted, air-dried cord blood sample showed lower G6PD activity measurement hence is not suitable for G6PD enzyme activity determination. 

## Acknowledgements

We are grateful to the families of the newborns for allowing us to use their newborn samples. Our appreciation goes to the Dean of the Faculty of Health Sciences, UiTM for providing budget, and Department of Diagnostic Laboratory Services, UKMMC for the research placement and instrumentations. We also would like to thank the Secretariat of Medical Research and Innovation, UKMMC for the ethics approval (Code Project: FF-2014-131) and Puan Ahlina in helping us with the manuscript.

## Conflict of interest

The authors declare no conflict of interest.

## Figures and Tables

**Table 1:**
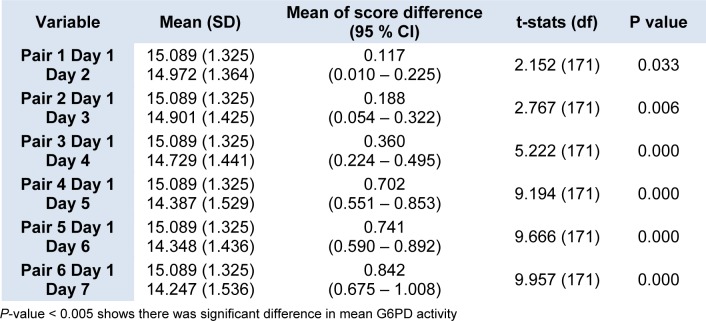
Daily mean G6PD activity of blood samples stored in EDTA tube from neonates with normal G6PD activity

**Table 2:**
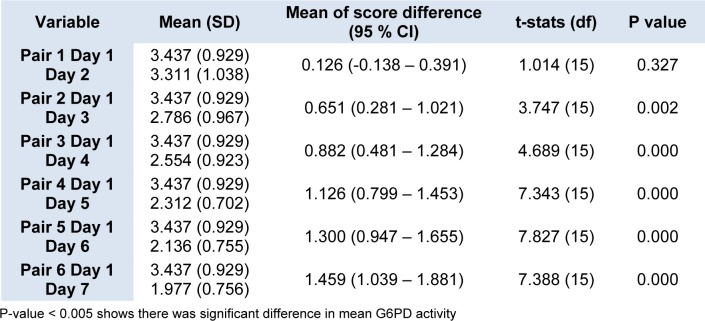
Daily mean G6PD activity of blood samples stored in EDTA tube from neonates with G6PD deficiency

**Table 3:**
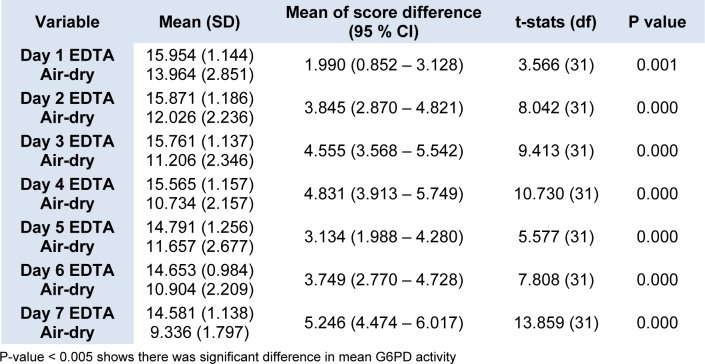
Comparison of daily mean G6PD activity between blood samples from EDTA tube and air dried blood spot on chromatography paper
